# The Grey Zones of Classic Hodgkin Lymphoma

**DOI:** 10.3390/cancers14030742

**Published:** 2022-01-31

**Authors:** Jan Bosch-Schips, Massimo Granai, Leticia Quintanilla-Martinez, Falko Fend

**Affiliations:** 1 Institute of Pathology and Neuropathology, Tübingen University Hospital and Comprehensive Cancer Center Tübingen-Stuttgart, 72076 Tübingen, Germany; jbosch@bellvitgehospital.cat (J.B.-S.); massimo.granai@med.uni-tuebingen.de (M.G.); Leticia.Quintanilla-Fend@med.uni-tuebingen.de (L.Q.-M.); 2 Department of Pathology, Hospital Universitari de Bellvitge—Bellvitge Biomedical Research Institute (IDIBELL), L’Hospitalet de Llobregat, 08907 Barcelona, Spain

**Keywords:** classic Hodgkin lymphoma, grey zone lymphoma, mediastinal large B cell lymphoma, nodular lymphocyte predominant Hodgkin lymphoma, diffuse large B cell lymphoma, gene expression profiling, immunohistochemistry, mutational analysis

## Abstract

**Simple Summary:**

Classic Hodgkin lymphoma (CHL) is a well-defined lymphoid neoplasm with a minority of characteristic neoplastic cells of B cell origin, namely Hodgkin and Reed–Sternberg cells immersed in a rich reactive inflammatory infiltrate in the background. Although CHL has always been set apart from non-Hodgkin lymphomas, cases with morphological and phenotypic features intermediate between CHL and other lymphomas have been described. Whereas some of these lymphomas only represent morphological mimics, others exhibit mutational and gene expression profiles which overlap with CHL, indicating that these cases, frequently termed grey zone lymphomas, reside on the biological boundary between CHL and large B-cell lymphomas. In the present review, we aim to describe the current knowledge of these rare lymphomas, address diagnostic issues and summarize today’s concepts on the classification of grey zone lymphomas and related tumors.

**Abstract:**

Classic Hodgkin lymphoma (CHL) is a well-defined neoplasm characterized by the presence of a minority of pathognomonic Hodgkin and Reed–Sternberg (HRS) cells in a reactive inflammatory background. Although genotypically of B cell origin, HRS cells exhibit a downregulated B cell program and therefore are set apart from other B cell lymphomas in the current WHO classification. However, cases with morphological and phenotypic features overlapping with CHL have been recognized, and the category of B cell lymphoma—unclassifiable—with features intermediate between diffuse large B cell lymphoma (DLBCL) and CHL, also termed grey zone lymphoma, was first introduced into the WHO classification in 2008 as provisional entity. These cases, as well as others raising a differential diagnosis of CHL can present diagnostic problems, as well as therapeutic challenges. Whereas some of these lymphomas only represent biologically unrelated morphological mimics, others, especially mediastinal grey zone lymphoma, exhibit genetic and gene expression profiles which overlap with CHL, indicating a true biological relationship. In this review, we address areas of diagnostic difficulties between CHL and other lymphoma subtypes, discuss the biological basis of true grey zone lymphoma based on recent molecular studies and delineate current concepts for the classification of these rare tumors.

## 1. Introduction

Classic Hodgkin lymphoma (CHL) is a clonal lymphoproliferative disease derived from germinal center B-cells, accounting for 15–25% of all malignant lymphomas [1,2,3,4]. Histopathologic diagnosis of CHL is based on the observation of scant mononuclear Hodgkin and multinucleate Reed–Sternberg (HRS) cells in an appropriate inflammatory background of reactive non-neoplastic cells, generally composed of varying numbers of small lymphocytes, eosinophils, neutrophils, histiocytes and plasma cells [5]. The paucity of neoplastic cells within the tumor, combined with an immunophenotype characterized by the downregulation of most B cell markers, made lineage identification challenging, and it required analysis of single microdissected HRS cells to confirm that they were clonal and of B cell lineage [6,7,8,9,10,11]. The 2017 revised World Health Organization (WHO) lymphoma classification recognizes four histological subtypes: nodular sclerosis CHL, lymphocyte rich CHL, mixed cellularity CHL and lymphocyte depleted CHL [5]. These subtypes are discerned mainly by architectural features, the composition of the background population and the tumor cell density, and differ in the frequency of EBV association, as well as in epidemiology and clinical presentation [7].

Although CHL can usually be separated reliably from other lymphomas based on morphology and immunophenotype, some cases exhibit overlapping features with non-Hodgkin lymphomas (NHL) or nodular lymphocyte predominant Hodgkin lymphoma (NLPHL) [12,13,14]. This is not surprising, given the B cell origin of CHL. A different composition of the background population, a higher proportion and a more monotonous appearance of HRS cells and an aberrant immunophenotype can all contribute to diagnostic difficulties. The recent elucidation of the genetic landscape and gene expression profile of CHL provided evidence that there is a true biological continuum with large B cell lymphomas, especially primary mediastinal large B cell lymphoma (PMBL), and suggests that cases with features intermediate between CHL and PMBL represent a true biological overlap, rather than just morphological mimics [15,16,17,18]. On the other hand, some biologically unrelated neoplasms, especially some DLBCL and T cell lymphomas may present morphological and phenotypic characteristics, which raise the differential diagnosis of CHL.

This review tries to summarize the current knowledge on the true biological “grey zones” of CHL and to delineate diagnostic criteria for lymphoma cases, which show overlapping morphological and immunophenotypic features with CHL, independent of the cell lineage. Of note, the term “grey zone lymphoma” should be restricted primarily to cases located in the mediastinum. For cases with features which overlap with CHL in other locations, efforts should be undertaken to assign them to one of the recognized entities in the current WHO classification, with mention of any unusual features. An indiscriminate use of the term grey zone lymphoma should be avoided [19]. The following topics will be addressed: mediastinal lymphomas, including primary mediastinal large B-cell lymphoma (PMBL) and so-called mediastinal grey zone lymphoma, cases intermediate between CHL and DLBCL in non-mediastinal location; NLPHL with unusual immunophenotype and overlap with CHL; EBV-associated B-cell lymphoproliferative diseases which may exhibit Hodgkin-like features, including EBV-positive DLBCL (EBV+ DLBCL) and mucocutaneous ulcer; and cases closely resembling CHL with T cell or cytotoxic phenotypes (Table 1).

## 2. Mediastinal Grey Zone Lymphoma (mGZL) and Related Entities

The anterior mediastinum is a common seat of two B-cell lymphoma entities—CHL of nodular sclerosis subtype and primary mediastinal (thymic) large B-cell lymphoma (PMBL) [20,21]. Based on the observation that cases with intermediate morphological and phenotypic features can arise in the mediastinum, the 2008 WHO classification introduced the provisional category of B-cell lymphoma, unclassifiable with features intermediate between DLBCL and CHL, to encompass cases that do not fit into either entity but show transitional features [5]. This concept was supported by studies demonstrating a close relationship between CHL and PMBL based on phenotypic similarities, the mutational landscape and gene expression profile, explaining cases with intermediate characteristics [15,16,17,22,23,24,25].

**CHL, nodular sclerosis (CHL NS),** the most frequent subtype of CHL (70% of the total) in USA/Europe, is characterized by collagen bands that delimit cellular nodules with HRS cells of the lacunar variant, and an accompanying inflammatory infiltrate composed of lymphocytes, eosinophils, histiocytes, and plasma cells [4,26]. Cases with numerous lacunar variant HRS cells grouped in aggregates or sheets have been designated syncytial variants of CHL NS [27,28]. The classic immunophenotype encompasses positivity for CD30, CD15 (in approximately 75–85% of cases), MUM1, weak PAX5, absence or only weak and heterogeneous expression of B cell antigens including CD20, CD79a and the B cell transcription factors BOB.1 and Oct-2 [5]. Of note, strong expression of a single B cell marker such as CD20 should not automatically trigger an inclusion into the GZL category [29]. In CHL NS, EBV is positive only in a minority of cases (10–25%). CHL NS predominantly affects young individuals in their 20s and 30s, slightly more women than men and usually presents in early stages (Ann Arbor I–II). Mediastinal involvement is common [5].

**PMBL** is a specific subtype of large B cell lymphoma thought to be of thymic B cell origin. It accounts for approximately 2–4% of NHL [17,30,31,32]. PMBL is characterized by a diffuse proliferation of medium to large cells with frequently clear cytoplasm, sometimes resembling lacunar cells. In addition, HRS cells may be observed [30,33,34]. There is diffuse compartmentalizing background sclerosis rather than nodule formation as in CHL NS. PMBL expresses pan-B cell markers including CD20, CD79a, PAX5, Oct-2, and BOB.1. In addition, it shows frequent positivity for CD23 (70%) and MAL, a highly specific marker for PMBL [23]. PMBL is variably positive for CD30 in >80% of cases and generally negative for CD15. Most cases express MUM1 and BCL6, while CD10 is rarely positive. EBV positivity is generally considered to exclude a diagnosis of PMBL [35]. Most PMBL cases lack immunoglobulin and MHC class I expression, the latter as evidence for active immune escape [36]. It predominantly affects young adults, with a male to female ratio of 1:2. Patients with PMBL frequently present with mediastinal bulk and may have superior vena cava syndrome or dyspnea and cough. PMBL shows rare nodal or bone marrow involvement, although it may affect cervical lymph nodes and uncommon extranodal sites, the latter especially in relapses. PMBL is more aggressive than CHL, although it exhibits a better prognosis than mGZL. Rare cases of large B-cell lymphoma without mediastinal involvement, but similar morphology, immunophenotype and/or gene expression profile as PMBL have been reported [17,37,38].

### 2.1. B-Cell Lymphoma, Unclassifiable, with Features Intermediate between Diffuse Large B-Cell Lymphoma and Classical Hodgkin’s Lymphoma (Grey Zone Lymphoma, GZL)

Conceptually first presented in 1998 [14], described in more detail in 2005 [16] and later incorporated as a provisional entity in the 2008 WHO classification, as unclassifiable B cell lymphoma with features intermediate between DLBCL and CHL, GZL remains a diagnostic and therapeutic challenge today. Although primarily applied to mediastinal lymphoma cases with morphological and phenotypical features intermediate between CHL and PMBL—so called mediastinal grey zone lymphoma (mGZL)—the concept became more broadly applied and the term “grey zone lymphoma” was also used for cases showing overlap with other B-NHL types. However, based on recent mutational and gene expression data, it seems that cases without mediastinal presentation are more heterogeneous and more closely related to DLBCL, sharing with mGZL mainly the commonality of overlapping morphological and phenotypical features to CHL [17].

#### 2.1.1. Mediastinal (Thymic Niche) Grey Zone Lymphoma

**Figure 1:**
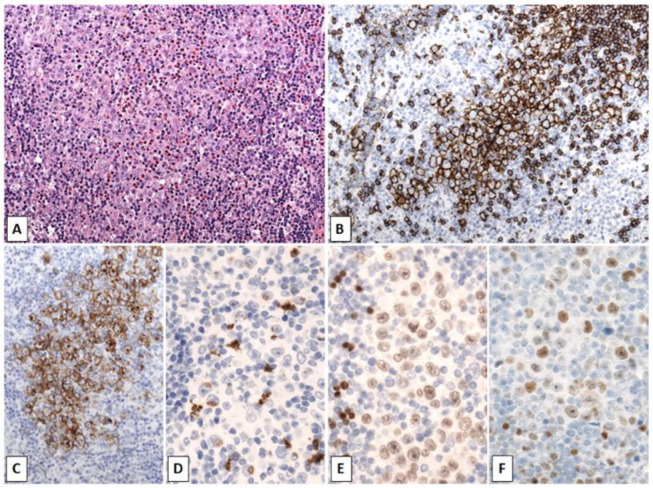
Mediastinal grey zone lymphoma. Mediastinal mass in a 16-year-old female with a nodular growth of relatively monotonous large cells with some lacunar cells and eosinophils (**A**). Strong positivity for CD20 (**B**) and CD30 (**C**). CD15 was focally weakly positive in a perinuclear dot-like fashion (**D**). Relatively weak expression of PAX5 (**E**), Oct-2 (**F**) and heterogeneous expression of CD79a (not shown). (Original magnification: (**A**) ×100, (**B**,**C**) ×200, (**D**–**F**) ×400).

Though mGZL most commonly affects young men averaging 30 years of age, it can also be observed in children [39]. It usually presents in early stages with mediastinal location and occasionally with supraclavicular lymphadenopathy, but bulky disease. Furthermore, mGZL presents a morphologic and immunohistochemical continuum with CHL and PMBL, but characteristically with more cellular pleomorphism and less accompanying inflammatory infiltration (Figure 1). Thus, one extreme is formed by cases resembling CHL NS, but with a full B cell program including Oct-2 and BOB.1, and CD15 negativity, and the other by cases morphologically compatible with PMBL, but exhibiting loss of B cell markers and strong expression of CD30 and frequently CD15, with the remainder showing true morphological and phenotypical hybrid features. Most cases of mGZL have a clear B cell phenotype with expression of more than one B cell marker in a panel of CD20, CD79a, Oct-2, BOB.1 and PAX5. Rare cases of true mGZL show EBV positivity [17,40,41]. Its recognition as a distinct entity has been reinforced based on unique clinical and genetic features, as well as by its inferior survival rates compared to CHL, PMBL or DLBCL.

#### 2.1.2. Gene expression profile, genetics and epigenetics of CHL, PMBL and mGZL

Gene expression studies, investigation of methylation profiles and mutational analysis performed by several groups have convincingly demonstrated that CHL NS, PMBL and mGZL are biologically related entities [15,17,24,25]. Although HRS cells have a loss of B cell program, they are B cells genotypically and carry somatically mutated and clonally rearranged IGH genes. On the genetic level, CHL NS, mGZL and PMBL share amplifications of 2p16—containing the *REL* oncogene—and 9p24—containing *JAK2* and the programmed-death ligand genes *PD1L1* and *PD1L2*. Translocations in the histocompatibility complex type II transactivator *CIITA* causing downregulation of MHC II are present in 15% of CHL cases and 38% of PMBL [25,42] and recurrent mutations occur in genes involved in the nuclear factor κB (NF-κB) pathway resulting in its constitutive activation.

Recent results by Sarkozy et al. [17] reinforced the notion that mGZL is a truly intermediate category between CHL and PMBL, with *SOCS1, B2M, TNFAIP3, GNA13, STAT6* and *NFKBIA* as the most frequently mutated genes in thymic niche mGZL, while generally lacking *BCL2* and *BCL6* translocations. In contrast, some of the highly mutated genes in DLBCL, such as *CARD11, MYD88, CD79B* (in activated B-cell type) or *EZH2* (in germinal center-type), were not observed in mGZL. Likewise, comparative gene expression profiling demonstrated an intermediate score for mGZL, between CHL and PMBL, with cell cycle- and extracellular matrix-related genes reflecting tumor cell density and the composition of the microenvironment [18]. Using large-scale methylation profiling of microdissected tumor cells and principal component analysis, Eberle et al. [25] documented that also epigenetically mGZL occupied a unique position, supporting the designation of a novel lymphoma category with intermediate features in relation to CHL and PMBL.

In summary, mediastinal CHL NS, mGZL and PMBL probably share their origin from thymic B cells and represent a clinical, biological and genetic continuum. They are jointly characterized by predominance in young adults with bulky mediastinal disease and frequent presence of immune escape mechanisms, and are discerned by distinct architectural features, variable numbers of reactive inflammatory cells and different levels of B cell marker expression. Table 2 summarizes clinical, morphological, phenotypical and genetic features of CHL, PMBL, mGZL and nmGZL.

#### 2.1.3. Non-Mediastinal Lymphomas with Grey-Zone Features

**Figure 2:**
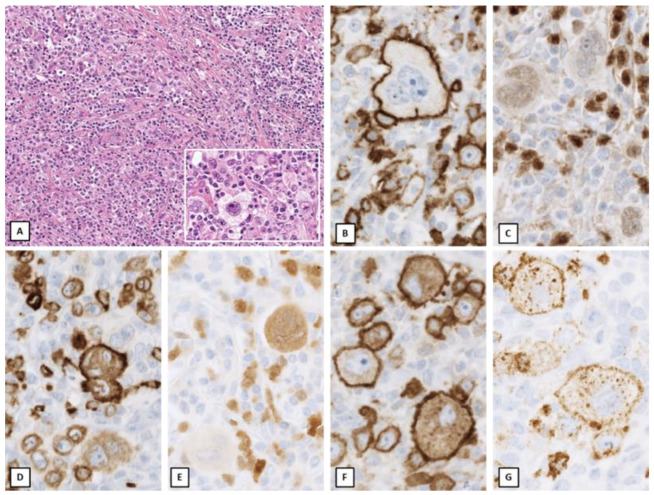
Non-mediastinal Grey Zone Lymphoma. Inguinal lymph node of a 48-year-old female patient. Architectural effacement with abundant pleomorphic HRS-like cells in a background of small lymphocytes, histiocytes and focal fibrosis ((**A**); A inset). The large cells show positivity for CD20 (**B**), PAX5 (**C**), CD79a (**D**), BOB1 (**E**), CD30 (**F**), CD15 (**G**). The HRS-like morphology of the neoplastic cells with co-expression of B cell markers as well as CD30 and CD15 best fit with a diagnosis of non-mediastinal grey zone lymphoma, although these cases genotypically are distinct from mGZL (Original magnification: (**A**): ×100, A inset: ×400; (**B**–**G**): ×600).

Although initially based on the observation of mediastinal cases, the definition of B-cell lymphoma, unclassifiable, with features intermediate between DLBCL and CHL (grey zone lymphoma) was not restricted to this location [13,43]. However, recent work has highlighted clinical, morphological and genetic differences between mediastinal and extramediastinal localizations. In contrast to its mediastinal counterpart, non-mediastinal GZL (nmGZL) typically presents in older women (mean age 55 years) with advanced clinical stages, with either nodal or extranodal involvement [44]. As in mGZL, these cases show a spectrum of morphology and immunophenotype, with variable presence of HRS-like cells and an increased inflammatory background and variable loss of B cell markers accompanied by expression of CD30 and/or CD15 (Figure 2). EBV positivity in cases with grey zone features should prompt inclusion in the EBV+ DLBCL category. Most cases of nmGZL exhibit a genomic profile distinct from mGZL. Sarkozy et al. [17] demonstrated that nmGZL are heterogeneous with characteristic mutations in apoptotic pathway genes that cluster into two subsets: a first subset with mutations of *TP53, BIRC6*, genes related to germinal center-derived DLBCL (*KMT2D, CREBBP, BCL2*), and *BCL2* or *BCL6* translocations, indicating a close relationship to DLBCL of germinal center type; and a second subset with mutations of *SOCS1* or *STAT6*, usually mutually exclusive with the first subset. On the transcriptional level, nmGZL has a gene expression profile more closely related to DLBCL.

A major confounding factor when studying GZL is the fact that diagnostic criteria are not well defined and variably interpreted, resulting in a low rate of consensus. In a study of 68 cases from 15 academic centers in North America, only 26 were confirmed as GZL upon review, the remaining were most often re-classified as CHL NS, followed by DLBCL, EBV+ DLBCL, PMBL and other CHL subtypes [29]. Main features of the confirmed GZL cases were an abundance of neoplastic cells with reduced inflammatory background and positivity for ≥ 1 B cell marker, cases re-classified as CHL NS showed more nodularity and were often rich in tumor cells, corresponding to NS grade 2 (10/27 cases), but frequently were strongly CD20+. The cases accepted as GZL more often had mediastinal disease (69% vs. 41%). However, despite the difficulties in classification there were no significant differences in disease-free and overall survival in this small retrospective series. When comparing the conclusions of this study with publications by other groups, it becomes evident that many GZL cases from other series would be classified differently by these criteria. For example, Sarkozy et al. have subdivided GZL into three groups based on morphology and phenotype, with CHL NS-like morphology characterized by marked nodular aggregates of HRS cells with variable perinodular fibrosis and occasional necrosis, but preserved B cell program (group 0), cases with PMBL-like morphology with a diffuse proliferation of large cells and clear cytoplasm, but downregulation of B cell markers (group 2) and cases with truly intermediate features (group 1) [17,44]. However, cases of group 0 likely would have been reclassified as CHL NS with strong B cell marker expression in the consensus study mentioned above [29].

## 3. Nodular Lymphocyte Predominant Hodgkin Lymphoma with Phenotypic Overlap with CHL

**Figure 3:**
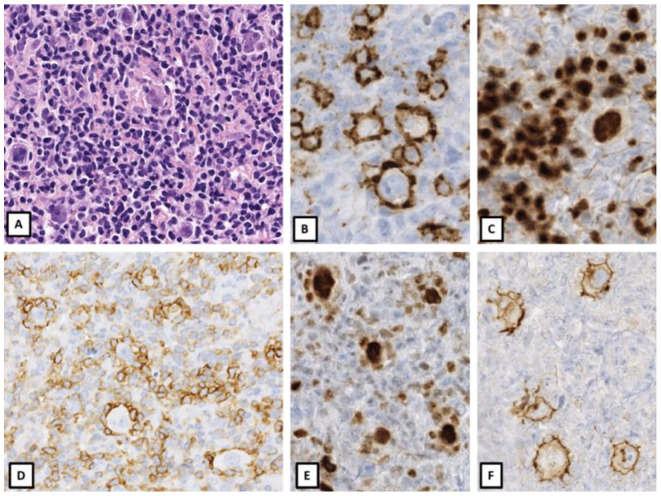
CD30+ Nodular lymphocyte predominant Hodgkin lymphoma. Enlarged axillary lymph node of a 63-year-old male patient. Nodal infiltrate of prominent LP cells in a background of small lymphocytes (**A**) strongly positive for CD20 (**B**) and PAX5 (**C**). PD1 positive T helper lymphocytes (**D**) rosetting around BCL6 positive LP cells (**E**). LP cells exhibit CD30 positivity (**F**). The prominent expression of B cell markers and the PD1+ rosettes exclude a diagnosis of CHL despite CD30 expression. (Original magnification: (**A**): ×400; (**B**–**F**): ×600).

**Figure 4:**
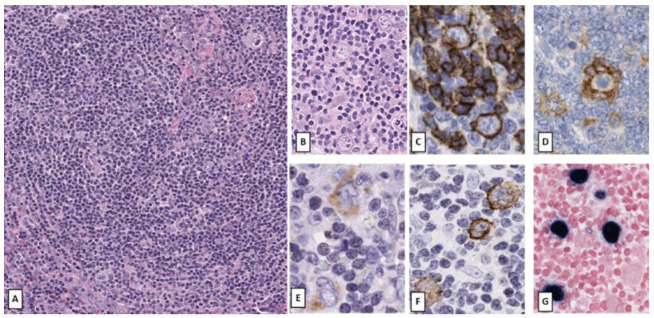
Epstein–Barr virus-positive nodular lymphocyte predominant Hodgkin lymphoma. Enlarged cervical lymph node of a 40-year-old female patient. Nodular growth pattern with PTGC-like follicles dominated by small lymphocytes (**A**) with scattered LP cells (**B**) which show a strong positivity for CD20 (**C**). PD1 positive follicular T helper lymphocytes (**D**) rosetting around LP cells, which variably express CD30 (**E**). LMP1 (**F**) and EBERs (**G**) positivity confirms association with EBV. (Original magnification: (**A**): ×200; (**B**): ×400; (**C**–**G**): ×600)].

Nodular lymphocyte predominant Hodgkin lymphoma (NLPHL) is a subtype of HL that accounts for approximately 5–10% of all cases of HL. NLPHL has usually an indolent course but with frequent late recurrences, more reminiscent of indolent B-NHL [45,46,47,48]. Histologically, NLPHL is characterized by the presence of large atypical lymphoid cells termed lymphocyte predominant cells (LP cells) or ‘popcorn cells’, arranged centrally or at the periphery of nodules of small B lymphocytes, often accompanied by epithelioid histiocytes, and with a notable absence of eosinophils and granulocytes. In addition to the classic nodular pattern reminiscent of progressively transformed germinal centers, cases with a predominantly diffuse growth pattern can occur. Fan et al. described six histologic growth patterns in NLPHL with distinct prognostic implications [49]. LP cells have scant cytoplasm and folded or multilobated nuclei and small to medium-sized basophilic nucleoli, but cells more resembling conventional HRS cells may be observed. LP cells show germinal center B cell immunophenotype with an intact B cell program and consistently express CD20, CD79a, CD45 and transcription factors PAX5, BCL6, Oct-2 and BOB.1 [50]. They also may show positivity for EMA in about half of the cases and may express IgD [41], the latter associated with B cell receptors recognizing antigens of Moraxella catharrhalis [51,52]. The background in the classical patterns according to Fan et al. is dominated by small B cells. LP cells are rosetted by small T lymphocytes with follicular T helper immunophenotype, with expression of CD3, CD4, PD1 and CD57 [53,54] (Figure 3 and Figure 4). At a genomic level, ultramicrodissection studies in LP cells have identified a high frequency of recurrent mutations in *JUNB, DUSP2, SGK1, SOCS1* and *CREBBP* [55,56].

### Differential Diagnosis with CHL

Morphologically, NLPHL shows similarities to lymphocyte rich CHL, which was separated from NLPHL mainly by the work of the German Hodgkin lymphoma study group [9] also usually exhibits a nodular growth pattern with dominance of small B-cells. However, the B cell nodules in CHL LR frequently contain small atrophic germinal centers, and the HRS cells, which sometimes can resemble LP cells but exhibit the classic CHL phenotype, are embedded in the expanded mantle zones. Diagnostic problems can arise in the differential diagnosis between NLPHL and CHL LR when there is aberrant expression of markers.

On one hand, HRS cells in CHL LR more frequently express B cell transcription factors such as Oct-2, BOB.1 and BCL6 than in other CHL subtypes [57]. Furthermore, a follicular T cell microenvironment with rosetting of PD1+ or CD57+ cells can be found in up to 50% of cases, leading some authors to postulate that CHL LR could represent an entity between CHL and NLPHL. In support of the existence of a grey zone between CHL and NLPHL, cases with documented clonal relationship between synchronous NLPHL and CHL have been reported [58].

On the other hand, LP cells may show expression of CD30 or CD15 and may exhibit EBV infection, features normally associated with CHL. Although most CD30+ cells in NLPHL were shown to represent reactive, clonally unrelated immunoblasts rather than LP cells using single cell analysis, faint to strong CD30 positivity in the neoplastic LP cells has been reported in up to 10% of NLPHL cases in some series, but no differences in clinical or histological features were noted [59]. CD30 expression in LP cells is not considered to be an adverse prognostic marker and is not associated with decreased overall survival [60]. CD15 expression has been typically used as a criterion of exclusion for NLPHL, and its positivity prompts reconsideration of CHL LR. However, there are rare cases of CD15+ NLPHL (6% of all NLPHL in one series) with classic morphology and preservation of B-cell immunophenotype [61]. These cases lacked expression of both CD30 and EBERs, but were positive for CD20, PAX-5, Oct-2, CD79a and BCL6; thus confirming the diagnosis of NLPHL. In contrast to CHL, NLPHL is traditionally considered EBV negative, but in a series encompassing both adults and children, around 4% of NLPHLs were found to be positive for EBV in the neoplastic LP cells [62]. These NLPHL cases had preserved expression for CD20 and Oct-2, but atypical weak expression for PAX5 and CD79a. In addition, cases also presented atypical morphological features for NLPHL, such as capsular fibrosis and atrophic germinal centers. All cases lacked CD15. CD30 expression was variable but was more commonly expressed in EBV+ NLPHL as compared to negative cases [63].

In summary, most cases of NLPHL and CHL LR can be accurately assigned to one of the two categories. NLPHL should exhibit a preserved B cell expression program, usually with strong co-expression of CD20, Oct-2 and BOB.1 and BCL6 positivity, and a follicular T cell background, whereas CHL LR usually shows expression of CD30 and CD15 and a variable downregulation of the B cell program. In addition to the markers mentioned above, expression of CD45, although frequently difficult to assess, EMA, J-chain and myocyte enhancer factor 2B (MEF2B) are useful in differentiating NLPHL from CHL [64]. MEF2B is positive in NLPHL but negative in CHL. Following these guidelines, only rare cases of HL may remain difficult to assign to either diagnosis. Genetic analysis currently is not useful for this differential diagnosis, let alone for practical reasons due to the sparsity of tumor cells.

## 4. EBV-Associated B-Cell Lymphoproliferative Diseases with Hodgkin-Like Features

Epstein–Barr virus-driven B-cell lymphoproliferative disorders (B-LPD) show a wide range clinical behavior, ranging from indolent, self-limiting disorders to aggressive lymphomas. Infection with EBV can cause morphological and phenotypical changes in B cells resulting in an HRS-like appearance. Therefore, CHL enters the differential diagnosis in many EBV+ B-LPD, including infectious mononucleosis and lymphoproliferations in immunosuppressed individuals. Below, EBV+ DLBCL and mucocutaneous ulcer will be discussed in more detail, since they may cause problems in separation from EBV+ CHL.

### 4.1. EBV-Positive Diffuse Large B-Cell Lymphoma (EBV+ DLBCL)

EBV+ DLBCL was first described in 2003 by Oyama et al. [65] as an aggressive EBV-positive lymphoma occurring in elderly patients in a setting of immunosenescence. It was a provisional entity in the 2008 WHO classification under the name EBV+ DLBCL of the elderly and was finally included in the 2016 update as a definitive entity under the name EBV+ DLBCL, NOS, since it was recognized that cases can also occur in younger patients [66,67]. EBV+ DLBCL occurs more commonly in males and has a worldwide distribution, although it is more frequent in countries with a higher prevalence of EBV-associated lymphoproliferative diseases, such as Asia or Latin America (7–15%) than in Western countries [65,68]. Whereas in elderly patients the majority of cases show that extranodal involvement affecting the gastrointestinal tract, lung and head and neck region and have a dismal prognosis, EBV+ DLBCL is a predominantly nodal disease in the younger age group, with 90% long term survival [66,69]. EBV+ DLBCL has a marked morphological variability, but its defining feature is the presence of large B cells of immunoblastic, centroblastic, or HRS-like morphology in an inflammatory background. EBV+ DLBCL can show a conventional DLBCL pattern; however, the majority of cases in young patients exhibit a T-cell and histiocyte rich large B-cell lymphoma-like or CHL-like morphology with large, atypical cells interspersed in an abundant reactive infiltrate. Necrosis and angioinvasion is common [70,71]. The neoplastic cells show an activated B cell phenotype, with CD20 expression in more than 50% of the cells [72] and frequent positivity for CD19, CD79a, PAX5, Oct-2, BOB.1, BCL6 and MUM1. They are usually positive for CD30 and have co-expression of CD15 in 23% to 68% of cases [69,73]. By definition, the majority of neoplastic cells are EBV positive, although a cutoff is not defined. The cells show predominantly EBV latency type II with expression of EBV latent membrane protein 1 (LMP-1) and less frequently (12–15%) type III with EBV nuclear antigen-2 (EBNA-2) positivity [69]. Activation of JAK/STAT and NF-kB pathways and a “host immune response” signature is detectable by gene expression profiling and suggests a virus-induced inflammatory microenvironment, with some overlap with EBV+ CHL. The mutational profile of EBV+ DLBCL is currently not well defined, and published works show little overlap in their results. Two recent studies have found recurrent mutations in *ARID1A, KMT2A/KMT2D, RHOA*, *CCR6*, *DAPK1*, as well as frequent 6q deletions [74,75]. Sarkozy et al. identified a profile distinct from mGZL, including EBV+ mediastinal cases, with an overall lower number of mutations than EBV− DLBCL and recurrent mutations in *STAT3* and structural alterations of *PDL2* indicating the importance of immune evasion [17,76].

**Figure 5:**
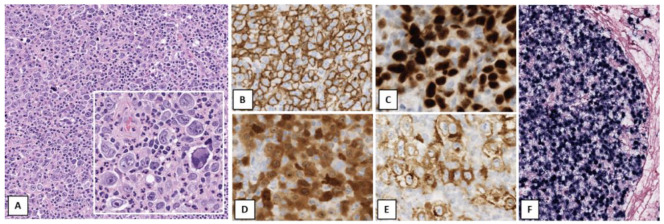
EBV+ Diffuse large B cell lymphoma with Hodgkin-like morphological features. Effaced supraclavicular lymph node in an 84-year-old male with large pleomorphic cells with HRS features (**A**). Diffuse positivity for CD20 (**B**), Oct-2 (**C**), BOB.1 (**D**) and CD30 (**E**). EBERs confirm EBV positivity (**F**). [Original magnification: (**A**): ×200, A inset: ×400; (**B**): 300×; (**C**–**E**): ×400; (**F**): ×100].

The differential diagnosis between EBV+ DLBCL with CHL-like features and EBV+ CHL can be challenging due to the morphological and phenotypical overlap [72]. Clinically, a primary extranodal manifestation speaks strongly against a CHL diagnosis. Morphologically, EBV+ DLBCL frequently shows a broader cytological spectrum of the HRS-like cells in a polymorphic background with more variation in the abundance of the reactive background population. Similar to the differential diagnosis between CHL and mGZL, the at least partial preservation of the B-cell program in EBV+ DLBCL with expression of CD20 in more than 50% of HRS-like cells, presence of B-cell specific transcription factors such as Oct-2 and BOB.1 are in favor of DLBCL, as well as the positivity for EBERs in variable large- to intermediate- and small-sized cells. Staining for LMP1, which is positive in most EBV+ DLBCL, highlights the variability of the EBV+ tumor cells, as well as the variability in LMP1 staining, whereas EBV+ CHL exhibits homogenous expression of LMP1 in the HRS cells. EBV latency type III with EBNA-2 positivity, observed in a minority of EBV+ DLBCL, is incompatible with a diagnosis of CHL [25]. The reactive background population contains a higher percentage of cytotoxic T cells in EBV+ DLBCL, and clonal T cell receptor rearrangements, an indication of clonal expansion of cytotoxic EBV-reactive T cells, in addition to clonal IGH rearrangements, are common in EBV+ DLBCL [70] (Figure 5).

### 4.2. Mucocutaneous Ulcer

Another EBV-driven lymphoproliferative disorder showing morphological and phenotypical overlap with EBV+ CHL, as well as EBV+ DLBCL, is EBV-associated mucocutaneous ulcer (MCU), which manifests as a localized EBV-positive ulcerated lesion in the context of frequently iatrogenic immunosuppression or immunosenescence in elderly patients. Lesions are typically located in oropharynx, gastrointestinal tract, and skin. MCU characteristically shows an indolent and self-remitting course. Management of MCU is conservative in most cases, including reduction or withdrawal of immunosuppressants. In some cases, lesion may undergo spontaneous resolution. Presence of lymph node involvement or systemic disease is incompatible with a diagnosis of MCU. In such cases, either a diagnosis of polymorphic EBV-associated lymphoproliferative disorder or EBV+ DLBCL should be considered, depending on the features of the case.

MCU was first reported by Dojcinov et al. [77] and was later recognized as a provisional entity in the 2017 WHO classification of lymphomas [78]. Histologically, it is characterized by well-defined ulcerations with EBV+ atypical B cells and a polymorphic inflammatory background with varying numbers of lymphocytes, histiocytes, plasma cells, and eosinophils. Characteristically, there is a rim of T lymphocytes present at the base of the ulcer, seemingly providing an immunological sequestration of the lesion. EBV+ lesional cells can reach a large size and may show immunoblastic and/or HRS-like features, and their number, shape, and arrangement can be variable, exhibiting distinct morphologic patterns which have been subclassified into four distinct groups: polymorphous (59%), large cell-rich (21%), CHL-like (12%) with numerous HRS-like cells and MALT-lymphoma-like (9%), with small-to-medium-sized EBV+ B cells and centrocytoid and plasmacytic features in the interfollicular zone [79]. However, this subtyping is not universally accepted. Immunophenotypically, MCU express, at least partially, B cell markers such as CD20, PAX5, and Oct-2, and are of activated phenotype. CD30 expression is constant, but half of the cases may also express CD15. EBERs are positive in a wide range of cell sizes. In clonality studies, less than half show Ig gene rearrangements and, in one-third of cases, TCR gamma rearrangements likely attributable to reactive clonal T expansions are identified. Separation of CHL-like cases of MCU from EBV+ CHL primarily relies on the clinical features with a localized oropharyngeal/gastrointestinal tract/skin ulceration in an elderly patient, frequently in the context of immunosuppression, often latency type III of EBV and demonstration of a partially preserved B cell phenotype in most cases.

## 5. Classic Hodgkin Lymphoma with Expression of T Cell Markers Versus ALK-Negative Anaplastic Large Cell Lymphoma

**Figure 6:**
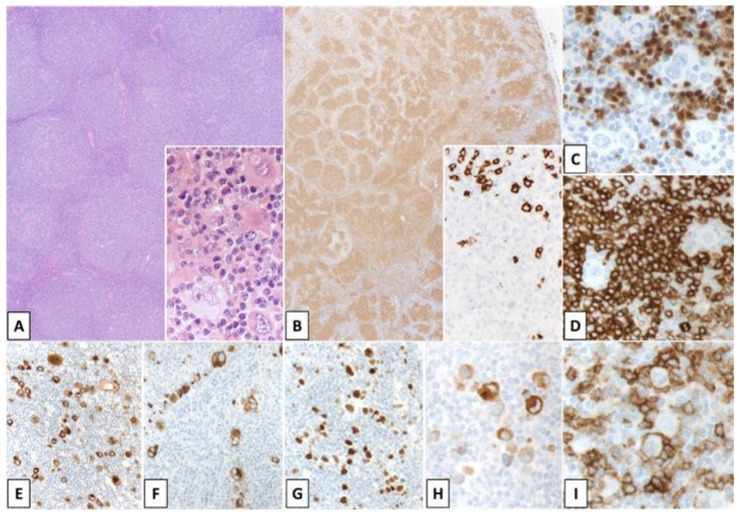
Lymphocyte rich CHL with expression of cytotoxic markers versus ALCL ALK- with CHL-like morphology. Enlarged inguinal lymph node of 3 cm in a 62-year-old male patient. Nodular growth pattern with HRS cells ((**A**); A inset). HRS cells are interspersed in nodules comprising CD20 positive small B lymphocytes (**B**), whereas the large cells show a clear CD20 negativity (B inset) as well as for other B-cell markers such as PAX5 (**C**) and CD79a (**D**). Positivity for CD30 (**E**), CD15 (**F**), and MUM1 (**G**) confirms the classic HL phenotype with an aberrant expression of cytotoxic markers as shown by perforin positivity (**H**). PD1 positive Tfh lymphocytes rosetting around HRS cells (**I**). [Original magnification: (**A**): 100×; A inset: 400×; (**B**): 50×; B inset: 200×; (**C**),(**D**),(**H**),(**I**): ×400; (**E**–**G**): ×200].

**Figure 7:**
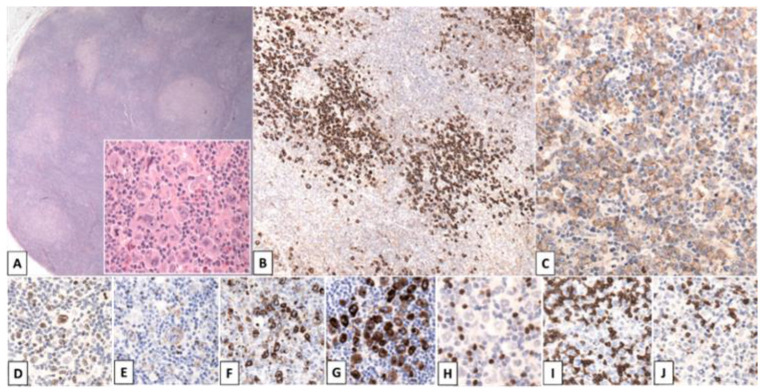
Cytotoxic Peripheral T-cell lymphoma NOS simulating CHL. Massive left-sided axillary lymphadenopathy in a 65-year-old male patient. Polymorphous infiltrate with nodular growth pattern (**A**) comprising prominent HRS-like cells (A inset). Positivity for CD30 (**B**), CD15 (**C**), MUM1 (**D**), TIA1 (**E**), Granzyme B (**F**) and Perforin (**G**). Clear negativity for PAX5 (**H**), CD3 (**I**) and CD8 (**J**). Molecular studies demonstrated a clonal TCR-γ rearrangement. [Original magnification: (**A**): 50×; A inset: ×400; (**B**): 100×; (**C**–**J**): ×200].

Although it is generally accepted that CHL is derived from activated germinal center cells B cells, also evidenced by attenuated expression of the B cell transcription factor PAX5, a subset of cases shows aberrant expression of T cell antigens in HRS cells [80,81] (Figure 6 and Figure 7). Tzankov et al. (2005) reviewed 259 cases of CHL and found aberrant T-cell marker expression in 10 cases (5%) [82]. CD2, CD4 and CD3 were the most expressed, followed by CD5 and CD8. No expression of CD7 was observed. Five cases (2%) expressed more than one T-cell antigen. Venkataraman et al. (2013) analyzed 50 cases of CHL with aberrant T-cell expression, the most commonly expressed antigens, in rank order, were CD2, CD4, CD5, CD7 and CD8 [83]. None of the cases tested positive for EBV, and no TCR rearrangements were detected. T cell antigen expression was associated with nodular sclerosis grade 2 histology and poor prognosis. In addition to T cell surface markers, the expression of cytotoxic molecules (perforin, granzyme B, and TIA1) has also been described in some CHL [81,84,85,86]. The largest series to date of 32 cases of CHL with cytotoxic phenotype has been compiled by Asano et al. [87]. They identified a male predominance with a mean age of 50 years. All cases expressed at least one of the cytotoxic markers on HRS cells, either TIA-1, granzyme B, or perforin. The neoplastic cells expressed CD30 and CD15, were positive for fascin and negative for CD45, and positive for EBV in up to 40% of cases. Of note, all cases were negative for PAX5 and CD20, and no IGH or TCR rearrangements were detected. The overall survival of patients with cytotoxic phenotype CHL-like after treatment was reduced compared to CHL. Whereas T cell antigen expression without demonstration of T cell clonality is considered an immunophenotypic aberrancy still compatible with a B cell genotype, only very rare cases with TCR rearrangements documented by single cell PCR have been reported. Although their classification has been a matter of controversy, cases with clonal T cell rearrangement probably should not be classified as CHL but as T-cell lymphomas with CHL-like features. [81,88]. Accordingly, recent reports have identified cases closely mimicking CHL morphologically and phenotypically with co-expression of CD30 and CD15, but lack of PAX5 and expression of cytotoxic granule proteins and presence of clonal TCR rearrangements. Huettl et al. documented two cases initially classified as CHL with cytotoxic marker expression recurring as PTCL NOS [89]. More recently, Fitzpatrick et al. described a series of CD30+ systemic T- cell lymphomas with anaplastic morphology and frequently CHL-like features, that harbored recurrent *JAK2* rearrangements [90]. In contrast to CHL, ALCL and other T cell lymphomas lack PAX5 expression, and the complete lack of PAX5 in a putative case of CHL should prompt more extensive phenotyping, including ALK protein and cytotoxic markers. However, it is of note that aberrant expression of PAX5 has been reported in ALK-negative anaplastic large cell lymphoma (ALCL), secondary to extra copies of the PAX5 gene [91,92].

Interestingly, the expression of T-cell transcription factors such as T-bet and GATA3 expression have also been reported in CHL [93,94]. T-bet, expressed in CD4+ T lymphocytes committed to Th1 T-cell development, is expressed in up to 80% of CHL and NLPHL [95]. GATA3, expressed in CD4+ T lymphocytes committed to Th2 T-cell development, is also expressed in CHL due to deregulation of NOTCH-1 and NFkB pathways [96], and can aid in the differential of CHL and other mediastinal lymphomas [22]. The exact oncogenic role of T-cell pathways in CHL is yet to be disclosed.

In summary, the expression of T cell markers in an otherwise typical case of CHL should not lead to reclassification as T cell lymphoma. However, cases with CHL with PAX5 negativity and significant expression of cytotoxic markers or T cell surface markers should be subjected to T cell clonality analysis, as the presence of a clonal T cell population would strongly argue against CHL. Nevertheless, the classification of PAX5 negative cases with CHL morphology and cytotoxic features as described by Asano et al. [87] remain controversial and will require more work on the genetic and gene expression profile.

## 6. Other Morphological Mimics of CHL

In addition to the situations described above, HRS-like cells can be observed in a variety of NHL subtypes, reactive disorders and immunodeficiency-associated lymphoproliferations without genetic or biological relation to CHL. In most such instances, the composition and immunophenotype of the background population allows a clear-cut discrimination from CHL by conventional morphology and immunophenotyping. Sometimes, such as in the case of peripheral T- cell lymphoma with HRS cells, molecular determination of clonality or even mutational analysis might be required to resolve the diagnosis (Table 3).

## 7. Conclusions

Clinicopathological studies of larger case series with detailed immunophenotyping and recent in-depth molecular studies of cases with overlapping features between CHL and large B-cell lymphomas have made it clear that this grey zone encompasses two distinct phenomena. First, mediastinal GZL, which shows a gene expression, methylation and mutational profile intermediate between PMBL and CHL, thus representing a true biological grey zone. Second, cases usually arising in extramediastinal locations, which show morphological and immunophenotypical similarities to CHL, but based on molecular and other findings are more closely related to DLBCL, EBV+ DLBCL or NLPHL, respectively. For diagnostic purposes, it should be kept in mind that a diagnosis of GZL should not be based on a single aberrant marker or morphological feature, but on the summary of clinical, morphological and immunophenotypical findings. The term GZL should be used sparingly and is usually restricted to EBV− mediastinal cases. Whether cases of true nmGZL and EBV+ mGZL exist is an area of active research.

## Figures and Tables

**Table 1:** The grey zones of classic Hodgkin lymphoma.

*B-cell lymphoma, unclassifiable, with features intermediate between diffuse large B-cell lymphoma and classical Hodgkin lymphoma (grey-zone lymphoma)* o Mediastinal grey-zone lymphoma o Non-mediastinal grey-zone lymphoma
*Nodular Lymphocyte-Predominant Hodgkin Lymphoma with unusual phenotype*
*EBV-associated B-cell lymphoproliferative diseases with Hodgkin-like features* o EBV-positive diffuse large B-cell lymphoma o Mucocutaneous ulcer
*Classic Hodgkin lymphoma-like lymphomas with expression of T- and/or cytotoxic markers*
**Mimicking Entities (Biologically Unrelated)**
Chronic Lymphocytic Leukemia and other indolent B-NHL with HRS-like cells Anaplastic Large Cell Lymphoma Angioimmunoblastic T-cell lymphoma and PTCL NOS with HRS-like cells Post-transplant and iatrogenic lymphoproliferative disorders with CHL-like morphology

**Table 2:** Comparison of clinico-pathological features of CHL, PMBL, mGZL and nmGZL.

	CHL	PMBL	mGZL	nmGZL
**Clinical** **Features**	Bimodal age distribution (15–35/50–70 y) Slight female predominance in NS majority in stage I/II Nodal involvement and frequent mediastinal bulk in NS	Young adults (median age 35 y), female predominance (2:1) Bulky mediastinal presentationRare extramediastinal nodal, extranodal or BM involvement	Young adults (mean age 32–37 y) with male predominance (1.4:1) Mediastinal bulk with nodal involvement More aggressive clinical course than PMBL and CHL	Equal sex distribution (mean age 55–61 y) 50% present in advanced stage Nodal or extranodal involvementEBV+ cases excluded
**Morphology**	HRS cells with abundant inflammatory infiltrate of lymphocytes, eosinophils, histiocytes, and plasma cells In NS collagen bands delimiting cellular nodules of HRS cells (lacunar variant) Tumor cell-rich syncytial variant	Diffuse nested infiltrate of medium-large mono-nuclear cells with frequently clear cytoplasm Delicate reticular fibrosis (“compartmental”) Lack of pleomorphism Occasional HRS-like cells Scant reactive infiltrate	Morphologic continuum with CHL and PMBL More cellular pleomorphism than PMBL and less reactive infiltrate than CHL Two extremes with discordance between morphology/phenotype: *CHL-like morphology* *PMBL-like morphology*	Morphologic continuum with CHL and DLBCL Marked pleomorphism with variable HRS-like cells, necrosis Variable inflammatory infiltrate Lack of collagenous nodular fibrosis
**Immunophenotype**	CD20−/+, CD79a−, Oct-2, BOB.1−, PAX5+ (weak) CD30+ (strong, homogenous), CD15+/−, MUM1+, BCL6− EBV −/+	CD20+, CD79a+, PAX5+, Oct-2+, BOB.1+, BCL6+, CD10−, CD30+ (weak expression), CD15−, CD23+ (70%), MAL+, MUM1+/− EBV−	CD20+/−, CD79a+/−, PAX5+ (strong), Oct-2+/−, BOB.1+/− (requires expression of > 1 B-cell marker) CD30+, CD15+ (*PMBL-like)*, CD15+/− (*CHL-like*) MUM1+, EBV− (rare +)	CD20+, CD79a+, PAX5+ (strong), Oct-2+/−BOB.1+/− (requires expression of > 1 B-cell marker), CD30+ CD15v, MUM1+ EBV− (otherwise classify as EBV+ DLBCL)
**Molecular** **Features and** **Genetics**	Loss of B cell expression programJAK/STAT and NF-kB activation Mutations in NFkB inhibitors (*TNFAIP3, NFKBIE, NFKB1A*) CN gains of *REL, JAK2* and *PD-L1/2* (9p24 amplification) Mutations in *JAK1/3, STAT3/5B/6, SOCS1* *CIITA* translocations	JAK/STAT and NF-kB activation CN gains of *REL, PD-L1/2* and *JAK2* (amplifications of 2p16 and 9p24) Loss of TNFAIP3, *NFKBIE* *EZH2, IL4R, GNA13* and *STAT6* mutations Activation of immune escape mechanisms	JAK/STAT and NF-kB activation CN gains of *REL, PD-L1/2* and *JAK2* Mutation in *SOCS1, B2M, TNFAIP3, GNA13* and *NFKBI* Lack of BCL2 and BCL6 translocations Mutational and gene expression profiles closer to PMBL/CHL	(Cluster 1) Mutations in *TP53, BIRC6, BCL2, KMT2D, CREBBP,* along with *BCL2* and *BCL6* translocations (Cluster 2) *SOCS1, STAT6* and/or *B2M* mutations Mutational and gene expression profiles closer to DLBCL

(+) nearly always positive; (+/−) majority positive; (−/+) minority positive; (−) (nearly always) negative; CHL NS—classic Hodgkin lymphoma nodular sclerosis subtype; PMBL—primary mediastinal large B-cell lymphoma; mGZL—mediastinal grey zone lymphoma; nmGZL—non-mediastinal grey zone lymphoma; DLBCL—diffuse large B-cell lymphoma; HRS cells—Hodgkin and Reed–Sternberg cells.

**Table 3:** Other morphological and phenotypical mimics of CHL.

Diagnosis	Main Features for Separating CHL
Chronic lymphocytic leukemia with HRS cells	Monotonous background population of small B cells with CD23 and CD5 expression, interspersed HRS cells are usually EBV+, may show partial preservation of B cell program and frequently lack CD15 [97,98,99]. HRS-like cells may also be observed in other small B-NHL.
Iatrogenic/Posttransplant lymphoproliferative disorders with CHL-like features	Extranodal presentation frequent. Usually polymorphic proliferation with interspersed immunoblasts and plasmablasts, variable amount of inflammatory reactive background population. HRS-like cells in EBV+ LPD frequently co-express CD20 and CD30, and are frequently associated with EBV latency type III, with EBNA2 expression [65,100,101,102,103,104].
Infectious mononucleosis (IM) and EBV reactivation	Tonsils and Waldeyer’s ring are usually affected in classic IM. Partial effacement of architecture with perifollicular presence of immunoblasts and plasmablasts with basophilic cytoplasm, small lymphocytes and frequently binucleate blasts closely resembling HRS cells. Frequent necrosis. Lack of CD15 expression and EBV type III latency in HRS-like cells, serological tests for EBV are positive for IgM antibodies [105,106,107,108,109,110,111]. Nodal presentation of IM and EBV reactivation with HRS-like cells may pose diagnostic difficulties.
Anaplastic large cell lymphoma ALK-	Usually diffuse or sinusoidal growth pattern, hallmark cells CD15−/+, PAX5-(rare cases positive), EMA+, cytotoxic markers +/−, T cell markers +/−. Clonal T-cell rearrangement [112,113,114].
Angioimmunoblastic T cell lymphoma with HRS-like cells	Polymorphous background population with clear cells, arborizing vessels and proliferations of follicular dendritic cells. HRS-like cells are usually EBV+ and can show variable B cell marker expression. Clonal T-cell rearrangement [115,116].
Peripheral T-cell lymphoma with HRS-like cells	High proliferative index in the accompanying small-medium cell component with frequent aberrant expression or loss of T-cell markers. HRS-like cells may co-express CD15 and C30 but lack PAX5. Clonal T-cell rearrangement [115,117,118,119].
